# Effect of Indoor Temperature on Physical Performance in Older Adults during Days with Normal Temperature and Heat Waves

**DOI:** 10.3390/ijerph14020186

**Published:** 2017-02-14

**Authors:** Ulrich Lindemann, Anja Stotz, Nina Beyer, Juha Oksa, Dawn A. Skelton, Clemens Becker, Kilian Rapp, Jochen Klenk

**Affiliations:** 1 Department of Clinical Gerontology and Rehabilitation, Robert-Bosch-Hospital, 70376 Stuttgart, Germany; anja.stotz@gmx.de (A.S.); clemens.becker@rbk.de (C.B.); kilian.rapp@rbk.de (K.R.); jochen.klenk@rbk.de (J.K.); 2 Musculoskeletal Rehabilitation Research Unit, Bispebjerg and Frederiksberg Hospitals, University of Copenhagen, 2400 NV Copenhagen, Denmark; ninabeyer.privat@gmail.com; 3 Quantified Employee, Finnish Institute of Occupational Health, 90220 Oulu, Finland; juha.oksa@ttl.fi; 4 Institute of Applied Health Research, Glasgow Caledonian University, Scotland G4 0BA, UK; dawn.skelton@gcu.ac.uk; 5 Institute of Epidemiology and Medical Biometry, Ulm University, 89081 Ulm, Germany

**Keywords:** adaptation, older adults, physical performance, indoor temperature

## Abstract

Indoor temperature is relevant with regard to mortality and heat-related self-perceived health problems. The aim of this study was to describe the association between indoor temperature and physical performance in older adults. Eighty-one older adults (84% women, mean age 80.9 years, standard deviation 6.53) were visited every four weeks from May to October 2015 and additionally during two heat waves in July and August 2015. Indoor temperature, habitual gait speed, chair-rise performance and balance were assessed. Baseline assessment of gait speed was used to create two subgroups (lower versus higher gait speed) based on frailty criteria. The strongest effect of increasing temperature on habitual gait speed was observed in the subgroup of adults with higher gait speed (−0.087 m/s per increase of 10 °C; 95% confidence interval (CI): −0.136; −0.038). The strongest effects on timed chair-rise and balance performance were observed in the subgroup of adults with lower gait speed (2.03 s per increase of 10 °C (95% CI: 0.79; 3.28) and −3.92 s per increase of 10 °C (95% CI: −7.31; −0.52), respectively). Comparing results of physical performance in absentia of a heat wave and during a heat wave, habitual gait speed was negatively affected by heat in the total group and subgroup of adults with higher gait speed, chair-rise performance was negatively affected in all groups and balance was not affected. The study provides arguments for exercise interventions in general for older adults, because a better physical fitness might alleviate impediments of physical capacity and might provide resources for adequate adaptation in older adults during heat stress.

## 1. Introduction

Excess outdoor temperature in the summer has resulted in an increased mortality during heat waves, especially among vulnerable older persons, in Europe [1,2] and worldwide [3,4,5]. At present, there are few recommendations on how to help older people adapt to these challenging conditions [6]. Understanding the association between temperature and relevant parameters of physical performance can inform recommendations on how to adapt health care and/or behavioral strategies at times of heat stress. 

Although outdoor temperature is the most effective parameter of climate condition, indoor temperature is even more relevant with regard to heat-related mortality and self-perceived health problems [7] and changes in physiological parameters, e.g., in blood pressure [8], in a population that spends much of its time indoors. At present, most knowledge about the effects of indoor heat on physical function has been gained using laboratory studies rather than studies conducted in the home environment. Studies investigating the effect of indoor temperature in the home environment of older people so far have not focused on physical performance [7,8,9]. When maximum performance is not necessary, a voluntary reduction in physical performance can be expected due to thermoregulatory behavior [10], but when maximum performance is tested, a reduced performance can be attributed to heat stress. With regard to maximum physical performance, a negative effect of high ambient temperature on endurance capacity is documented for young athletes [11,12] and older women in the laboratory [13,14]. In contrast, a moderate increase in muscle temperature improves short-duration exercise performance by an increase in all chemical reactions, nerve conduction and contractibility of muscle fibers [15]. Little is known about the effect of heat on physical performance in daily life, such as walking, standing up from a chair and balance, which is especially relevant for older adults in terms of fall risk [16,17,18]. Furthermore, it is unclear whether the association is different for short-duration exposure to heat compared to heat waves with supposed cumulative heat stress. 

The aim of this study was to describe the association between indoor temperature and physical performance in the home environment of older adults during the summer of 2015, which was one of the hottest ever in Germany with two heat waves in July and August.

## 2. Materials and Methods 

### 2.1. Subjects and Design

For this panel study with repetitive measurements, a convenient sample of independent older adults was recruited in 10 facilities of sheltered living in the city of Stuttgart in Southern Germany, one of the warmest regions in Germany with the warmest mean day-temperature of 23.3 °C in July. Inclusion criteria were an age of 60 years and older, no need of formal or informal support and a regular outdoor activity of at least 30 min per day. Exclusion criteria were not being able to follow instructions, uncontrolled cardiac illness, use of a wheel chair and terminal illness. All subjects gave their informed consent for inclusion before they participated in the study. The study was conducted in accordance with the Declaration of Helsinki, and the protocol was approved by the ethical committee of the University of Tuebingen (080/2015/BO2). 

### 2.2. Assessment Protocol

Starting in May 2015 and ending in October 2015 the participants were visited every four weeks. Additional assessments were conducted during two heat waves in July and August with a heat wave defined as three or more consecutive days with maximum outdoor temperatures of 30 °C or more and a heat alarm from the German Meteorological Service. Thus, a maximum of eight assessments were conducted per participant. The duration of each visit was approximately 30 min. All assessments were performed between 8 a.m. and 3 p.m. visiting each participant individually at the same time of the day.

### 2.3. Repetitive Assessments

#### 2.3.1. Climate Condition

Indoor temperature and humidity were measured during each visit with a data logger (HL-1D, ROTRONIC Messgeräte GmbH, Ettlingen, Germany), which was placed on a table in the shade in the room where the assessment was conducted. Since there was hardly any change of temperature and humidity (maximum 1 °C and 1%, respectively) during the assessment, the maximum values were used and a heat-index was calculated. 

#### 2.3.2. Physical Performance:

Balance was tested during unsupported standing for 10 s in consecutively more challenging positions if possible (open stance, closed stance, semi-tandem stance, tandem stance and one-leg stance, each with eyes open) for a maximum of 50 s in total. Here, a common used test of static balance [19] was modified in order to avoid possible floor and ceiling effects. Therefore, the lowest and highest performance measures (i.e., open stance and one-leg stance) were added. For the timed five-chair-rise test [19] the participants were instructed to stand up and sit down five times as fast as possible without using arm rests. Habitual gait speed was measured over a distance of 4 m. If needed, assistive devices could be used. All tests of physical performance were measured using a stopwatch and time and speed were used for analysis.

### 2.4. Descriptive Measures

Age, body mass and body height were recorded at the first visit and the body mass index was calculated.

Co-morbidity was assessed via a standardized questionnaire asking for 18 age-relevant diseases [20]. Yes/no answers resulted in a maximum (worst) score of 18. 

In order to create subgroups, frailty was assessed by the five criteria established by Fried et al. [21] (i.e., gait speed, hand grip strength, weight loss, exhaustion, physical activity), was assessed via the original measures and was scored 0–5 according to the number of fulfilled criteria. ‘Pre-frailty’ was present if one or two criteria were fulfilled and ‘frailty’ was present if three or more criteria were fulfilled. Since only 11 participants (13.6%) were frail according to these criteria with most participants categorized as pre-frail (*n* = 52; 64.2%) or not frail (*n* = 18; 22.2%), one of these criteria, i.e., low gait speed, was used as a possible effect modifier to create subgroups instead for further analyses. According to Fried et al. [21] the gender specific cut-offs for low gait speed were 0.653 m/s or slower for women and men with body height less than 159 cm and 173 cm, respectively, and 0.762 m/s or slower for women and men with body height of at least 159 cm and 173 cm, respectively. The rationale to use gait speed as a surrogate of frailty in this context was because it is highly correlated with frailty [22]. Furthermore, it has high face-validity and is the only frailty criterion to be assessed objectively with a ubiquitous tool (stop watch). Categorizing the participants according to gait speed sub-groups of 35 adults with initially lower gait speed and 46 adults with initially higher gait speed were created. 

Cognition was screened by the Short Orientation Memory Concentration test [23] with weighted scores of 0 (best) to 28 (worst) and a score of >10 suggesting cognitive impairment. 

### 2.5. Statistics

Based on overall distribution and in order to have enough cases in the lowest and highest category, for graphical description the results were grouped into five categories of corresponding room temperatures (<22 °C, *n* = 121; 22–23.9 °C, *n* = 156; 24–25.9 °C, *n* = 111; 26–27.9 °C, *n* = 97; >27.9 °C, *n* = 53). Least-square means were calculated for each of these groups using multilevel linear regression analyses, due to the hierarchical structure of the data with several repeated measurements (level 1) per person (level 2). To consider the correlation of repeated measurements, an unstructured covariance matrix was used. Multilevel linear regression models were also used to calculate the association between indoor temperature and the considered physical performance measures as well as to estimate the differences between days within and outside heat waves. Heat wave (within and outside) was treated as a dichotomous variable. All analyses were additionally stratified by gait speed to assess possible effect modification. All analyses were conducted using SAS version 9.4 software (SAS Inc., Cary, CA, USA). 

## 3. Results

Eighty-one older adults with a mean age of 80.9 years (68 women, 84%) were included. A total of 538 visits/assessments (mean 6.6 per person) were conducted, of which 111 visits/assessments (mean 1.6 per person) were conducted during the two heat waves in July and August. All missing visits were due to participants having other engagements. No visit was canceled in relation to perceived heat-stress. The minimum indoor temperature of all visits was 17.2 °C on 14 October, and the maximum indoor temperature was 30.3 °C on 7 July with only seven (1.3%) missing temperature values due to a device not working. During the assessment period, one participant died after the fifth visit and one participant moved to a nursing home after the fourth visit. Participants with an initially lower gait speed (*n* = 35) reported a higher number of diseases and symptoms (5.18; 95% CI: 4.49; 5.87 versus 3.39; 95% CI: 2.74; 4.04) and a higher number of drugs (6.11; 95% CI: 4.95; 7.27 versus 3.89; 95% CI: 2.99; 4.79) than those with an initially higher gait speed (*n* = 46). A description of the total group and sub-groups of participants with initially lower and higher gait speed is shown in detail in Table 1.

**Figure 1:**
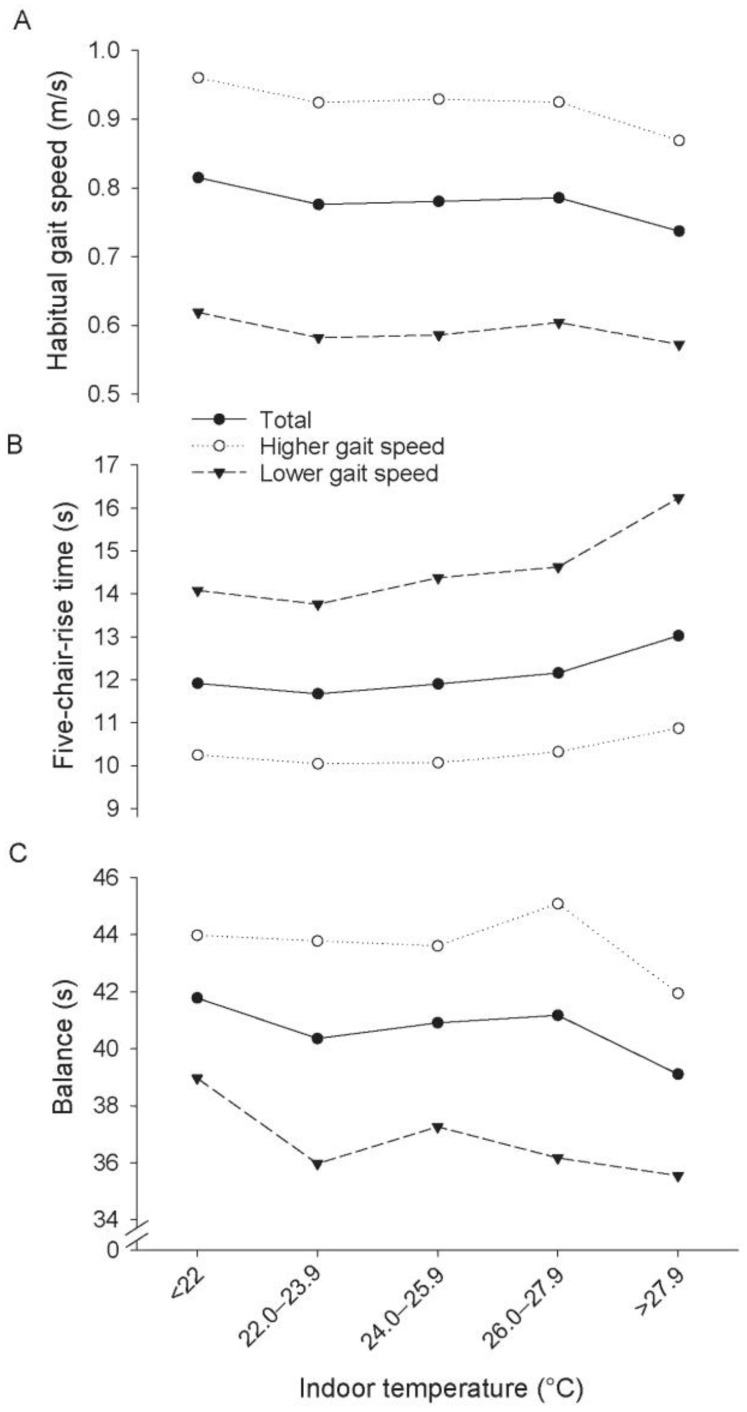
Least-square means from multilevel linear regression analysis of physical performance (habitual gait speed (A), chair-rise performance (B), balance performance (C)) related to grouped indoor temperature and stratified by gait speed (thresholds indication of low gait speed for body heights <159 cm (women)/173 cm (men) = 0.653 m/s and ≥159 cm (women)/173 cm (men) = 0.762 m/s).

Multilevel linear regression analysis revealed a negative effect of the indoor temperature on the habitual gait speed, chair-rise time and balance (Figure 1). Similar patterns were observed for the heat index, but are not presented because of the better usability of temperature.

A negative association between temperature and habitual gait speed was seen in the total group and also within the sub-group of adults with an initially higher gait speed. In this subgroup of adults with an initially higher gait speed, the effect was strongest with a reduction of the habitual gait speed from 0.96 m/s (95% CI: 0.90 m/s; 1.02 m/s) in the temperature category <22 °C to 0.89 m/s (95% CI: 0.80 m/s; 0.93 m/s) in the temperature category >27.9 °C. The detrimental effect of increased temperature on chair-rise time (longer time to rise five times) was seen in the total group and within both sub-groups of adults with initially higher and lower gait speeds. In the sub-group of adults with an initially lower gait speed, the effect was strongest, with an increase of performance time from 14.08 s (95% CI: 12.33 s; 15.83 s) in the temperature category <22 °C to 16.24 s (95% CI: 14.32; 18.15 s) in the temperature category >27.9 °C. However, the detrimental effect of an increase in temperature on balance was seen only in the subgroup of adults with an initially lower gait speed, with a decrease of performance time from 38.96 s (95% CI: 35.94 s; 41.99 s) in the temperature category <22 °C to 35.55 s (95% CI: 31.87; 39.23 s) in the temperature category >27.9 °C. 

The detrimental effect of an increase of the indoor temperature by 10 °C on the measures of physical performance and the differences of functional performance between heat waves and normal days are presented in Table 2. There was a statistically significant difference in the mean change per 10 °C between persons with higher and lower gait speeds (3.74 s, 95% CI: 0.93 s; 6.55 s). In general, similar associations and patterns were seen when comparing results from assessments made in the absence of a heat wave to those during a heat wave.

## 4. Discussion

This study shows that high indoor temperatures in an older adult’s own home negatively affect their physical capacity. Although the clinical relevance of the amount of decrease in the physical performance in our study may be low, all results point in the same direction. These results are in line with a laboratory-based study showing a reduced endurance capacity in the heat, documented for young athletes [11]. A decreased endurance capacity in a cohort of healthy older women has been also shown when a six-minute walk test was conducted after a short time exposure (45 min) in 30 °C conditions [14]. Although a moderate increase of temperature can increase muscle function [15] and physical activity [24], the extraordinary stress of cumulative heat during a heat wave showed that physical performance in older adults is decreased. Since the chair-rise performance and balance were tested as maximum performance tests (as fast as possible, as long as possible), our results can be seen as a negative effect possibly increasing the risk of falls. This assumption is supported by the evidence of poor performance in chair-rise time and balance, resulting in falls [17,18]. The effect was especially notable in those older adults with a limited physical performance, i.e., lower gait speed, who also have worse medical conditions, indicated by a higher number of co-morbidities and medications taken. This is in contrast to a voluntary reduction of performance during habitual behavior as a thermoregulatory effect [10]. 

Since physical capacity is a relatively modifiable parameter and better physical performance is also associated with better outcomes, such as lower fall risks [25], short-term mortality and nursing home admissions [19], these results suggest that interventions to improve physical fitness in general might alleviate the effects of heat stress on physical performance. 

Walking speed was assessed as the habitual gait speed, meaning the preferred speed. In the context of human thermoregulation, a voluntary decrease in habitual performance with increasing temperature might be seen as an adequate adaptation to heat stress [26,27], if the person has the capacity to do so and the maximum performance is not necessary. Within this study, older adults with an initially lower gait speed did not adapt, whereas those with an initially higher gait speed were able to adapt, suggesting that those who are less frail have some spare capacity to adapt. However, the confidence intervals for reductions in performance overlap, so the differences may not be significant. A trade-off between a necessary minimum walking speed for safety requirements in daily life and a reasonable adaptation might be positively influenced by exercise interventions aimed at improving gait speed and function. 

The results of this study cannot be generalized as this was a convenience sample, although they are in line with other studies including large samples of older adults [10]. Due to organizational reasons, the participants could not be visited at the same time of the day. Suggesting a direct association between temperature and physical performance, this fact was not regarded in the analyses. This practice was confirmed by comparable results during the heat waves. Nevertheless, future studies should also investigate a possible diurnal effect on physical performance. Environmental aspects of the buildings, such as thermal insulation or shading, were incongruent for the participants’ flats. Therefore, outdoor temperature could not be used for the analyses. Heat-adaptive behavior, such as opening windows, was not assessed but was reflected by the indoor temperature. If our study will be replicated in other cohorts and possibly in more active cohorts, the pre-test activity should be investigated as a possible effect modifier. Since most participants in this study were cognitively intact, this possible effect modifier could not be investigated. Future studies should investigate different cohorts, including cognitively impaired older adults, and should investigate other effect modifiers.

## 5. Conclusions 

This study has shown significant detriments to physical function in older adults during hot indoor temperature conditions. It provides a further argument for exercise interventions in general for older adults, as better physical fitness might alleviate the effects of heat stress and might provide extra physical reserves for adaptability.

## Figures and Tables

**Table 1:** Baseline characteristics of all participants.

		Habitual Gait Speed	
	Total (*n* = 81)	Low * (*n* = 35)	High * (*n* = 46)
Female, *n* (%)	68 (84)	28 (80)	40 (87)
Age (years), mean (SD)	80.9 (6.53)	81.6 (7.42)	80.4 (5.79)
Body height (cm), mean (SD)	158.7 (8.13)	157.5 (8.85)	159.5 (7.56)
Body Mass Index (kg/m^2^), mean (SD)	27.9 (4.75)	27.9 (4.16)	28.0 (5.18)
SOMC score (0–28), mean (SD)	3.51 (3.17)	3.41 (2.46)	3.58 (3.62)
Co-morbidity score (0–18), mean (SD)	4.15 (2.33)	5.18 (2.07)	3.39 (2.25)
Respiratory diseases, *n* (%) **	16 (20)	6 (17)	10 (22)
Cardiovascular diseases, *n* (%) **	33 (41)	19 (54)	14 (30)
Drugs (n), mean (SD)	4.85 (3.45)	6.11 (3.50)	3.89 (3.11)
Frailty score (0–5), mean (SD)	1.37 (1.08)	2.14 (0.94)	0.78 (0.76)
Non-frail, *n* (%)	18 (22)	0 (0)	18 (39)
Pre-frail, *n* (%)	52 (64)	25 (71)	27 (59)
Frail, *n* (%)	11 (14)	10 (29)	1 (2)
Habitual gait speed (m/s), mean (SD)	0.76 (0.25)	0.53 (0.12)	0.92 (0.20)
Balance (s), mean (SD)	40.5 (7.94)	36.6 (8.25)	43.2 (6.55)
Five-chair-rise time (s), mean (SD)	11.3 (3.42)	13.2 (3.54)	10.1 (2.76)
Total number of measurements, *n*	538	223	315
Number of measurements during a heat wave, *n*	111	45	66

* Frailty criteria for gait speed, i.e., slow speed: 0.653 m/s or slower for women/men with body height <159/173 cm, and 0.762 m/s or slower for women/men with body height ≥159/173 cm; ** assessed via co-morbidity questionnaire; SD: standard deviation; SOMC: Short Orientation Memory Concentration test; better score values are underlined; results of physical performance measures were taken from the first assessment in May at a mean indoor temperature of 22.7 °C.

**Table 2:** Mean changes of functional performance with increasing indoor temperature and mean differences of functional performance between heat waves and normal days.

Physical Performance; Group of Participants	*n*/Obs	Mean Change of Functional Performance Per 10 °C Increase of Indoor Temperature with 95% CI ^†^	Mean Difference of Functional Performance between Heatwaves and Normal Days * with 95% CI ^†^
Gait speed (m/s); all adults	81/537	−0.074 (−0.113; −0.034)	−0.041 (−0.065; −0.018)
Gait speed (m/s); adults with initially higher gait speed	46/315	−0.087 (−0.136; −0.038)	−0.062 (−0.092; −0.032)
Gait speed (m/s); adults with initially lower gait speed	35/222	−0.044 (−0.109; 0.021)	−0.010 (−0.048; 0.027)
Chair-rise (s); all adults	81/514	1.15 (0.58; 1.73)	0.90 (0.55; 1.24)
Chair-rise (s); adults with initially higher gait speed	46/313	0.67 (0.12; 1.23)	0.57 (0.23; 0.90)
Chair-rise (s); adults with initially lower gait speed	35/201	2.03 (0.79; 3.28)	1.44 (0.72; 2.16)
Balance (s); all adults	81/538	−1.69 (−3.56; 0.182)	−1.11 (−2.24; 0.01)
Balance (s); adults with initially higher gait speed	46/315	−0.18 (−2.33; 1.98)	−0.93 (−2.27; 0.41)
Balance (s); adults with initially lower gait speed	35/223	−3.92 (−7.31; −0.52)	−1.36 (−3.33; 0.61)

* Average indoor temperature on normal days = 23.2 (2.17) and on days during heatwaves = 27.6 (1.23); ^†^ multi-level linear regression models; *n* = number of subjects; Obs: number of observations; CI: confidence interval.
